# Neocentric X-chromosome in a girl with Turner-like syndrome

**DOI:** 10.1186/1755-8166-5-29

**Published:** 2012-06-09

**Authors:** Morteza Hemmat, Boris T Wang, Peter E Warburton, Xiaojing Yang, Fatih Z Boyar, Mohammed El Naggar, Arturo Anguiano

**Affiliations:** 1 Cytogenetics Dept, Quest Diagnostics Nichols Institute, San Juan Capistrano, CA, USA; 2 Deparment of Genetics and Genomic Sciences, Mount Sinai School of Medicine, NY, USA; 3 Quest Diagnostics Nichols Institute, 33608 Ortega Highway, San Juan Capistrano, CA, 92690, USA

**Keywords:** Neocentromere, Turner Syndrome, X-inactivation, Mosaicism

## Abstract

**Background:**

Neocentromeres are rare human chromosomal aberrations in which a new centromere has formed in a previously non-centromeric location. We report the finding of a structurally abnormal X chromosome with a neocentromere in a 15-year-old girl with clinical features suggestive of Turner syndrome, including short stature and primary amenorrhea.

**Result:**

G-banded chromosome analysis revealed a mosaic female karyotype involving two abnormal cell lines. One cell line (84% of analyzed metaphases) had a structurally abnormal X chromosome (duplication of the long arm and deletion of the short arm) and a normal X chromosome. The other cell line (16% of cells) exhibited monosomy X. C-banding studies were negative for the abnormal X chromosome. FISH analysis revealed lack of hybridization of the abnormal X chromosome with both the X centromere-specific probe and the “all human centromeres” probe, a pattern consistent with lack of the X chromosome endogenous centromere. A FISH study using an XIST gene probe revealed the presence of two XIST genes, one on each long arm of the iso(Xq), required for inactivation of the abnormal X chromosome. R-banding also demonstrated inactivation of the abnormal X chromosome. An assay for centromeric protein C (CENP-C) was positive on both the normal and the abnormal X chromosomes. The position of CENP-C in the abnormal X chromosome defined a neocentromere, which explains its mitotic stability. The karyotype is thus designated as 46,X,neo(X)(qter- > q12::q12- > q21.2- > neo- > q21.2- > qter)[42]/45,X[8], which is consistent with stigmata of Turner syndrome. The mother of this patient has a normal karyotype; however, the father was not available for study.

**Conclusion:**

To our knowledge, this is the first case of mosaic Turner syndrome involving an analphoid iso(Xq) chromosome with a proven neocentromere among 90 previously described cases with a proven neocentromere.

## Background

Neocentromeres are rare human chromosomal aberrations that have apparently formed within interstitial chromosomal sites that have not previously been known to express centromere function. An acentric fragment that would usually be lost can rescue itself by generating a neocentromere, which functions similarly to a normal centromere. Neocentromeres lack α-satellite DNA and have consistently demonstrated the presence of all centromere proteins except centromeric binding protein (*CENP-B*) [1].

As summarized by Liehr et al. [2], neocentric chromosomes are based on a U-type exchange and the formation of inverted duplicated chromosomes [2-4] or inverted duplications on acentric markers [5]. The resulting marker comprises two copies of the chromosome segment oriented as a “mirror image” around the breakpoint. Neocentromere formation occurs at an interstitial site apparently unrelated to the site of the breakpoint. However, the generation of the neocentromere allows the recovery of the acentric fragment that would otherwise have been lost and thereby restores a balanced karyotype [6]. Extensive analysis of neocentromere formation has led to the conclusion that neocentromere activation occurs via an unknown epigenetic mechanism that, in effect, converts a previously non-centromeric genetic locus into a functional neocentromere that associates with all of the proteins involved in active centromere function [6]. This process has recently been described as neocentromerization [7].

DNA polymorphism studies performed in five cases indicated that human neocentromeres can form either during meiosis [8,9] or mitosis [8]. Once formed, they can also be transmitted through mitosis and meiosis [5].

Mosaicism might be a consequence of mitotic instability of neocentric marker chromosomes that have been meiotically transmitted from the previous generation [10-12]. This would be due to either suboptimal function of the neocentric kinetochore or selection pressure against cells containing the marker [13]. Alternatively, mosaicism could arise from a meiotically derived marker if neocentromere function was not established at the time of meiotic rearrangement. In this scenario, neocentric function would develop after several post-fertilization cell divisions, during which some of the markers would be lost [14].

To date, more than 90 cases of neocentromeres involving 20 different human chromosomes have been described [15-24], including only two cases of neocentric X chromosome. Yu et al. reported a case with a supernumerary neocentric marker chromosome, which consisted of partial duplication of the short arm of X chromosome in 100% of G-banded metaphases [22]. The second case was mosaic for 45,X and 46,X,rec(Xq) with features of Turner syndrome [25]. We report here a patient with features of Turner syndrome who was mosaic for two cell lines, including 45,X and 46,X,i(Xq); the latter contained an active neocentromere and was monosomic for Xp and partially trisomic for Xq.

## Results

**Figure 1:**
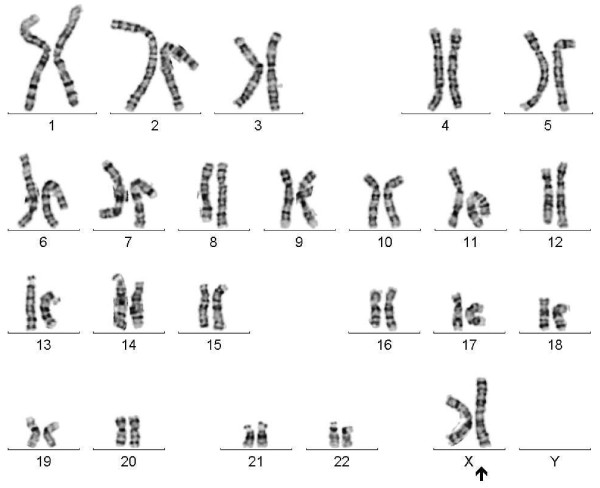
G-banded karyotype showing the cell line with one normal and one abnormal X chromosome with duplication of long arm and deletion of short arm.

**Figure 2:**
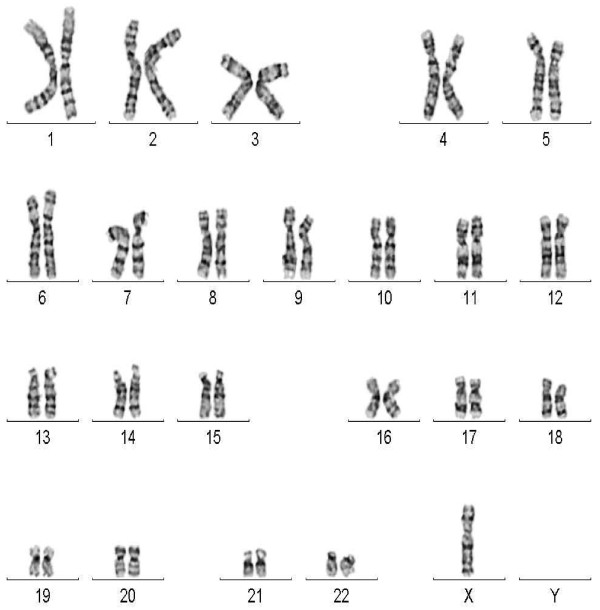
G-banded karyotype showing a cell line with monosomy X.

Chromosome analysis of cultured lymphocytes by G-banding revealed a mosaic female karyotype involving two abnormal cell lines. One cell line (84% of analyzed metaphases) had a normal X chromosome in addition to a structurally abnormal X chromosome with duplication of the long arm and deletion of the short arm (Figure 1). The other cell line (16% of cells) exhibited monosomy X (Figure 2). The proband’s mother had a normal female karyotype, 46,XX. The father was not available for karyotyping. However, the abnormal X is very likely to be de novo.

**Figure 3:**
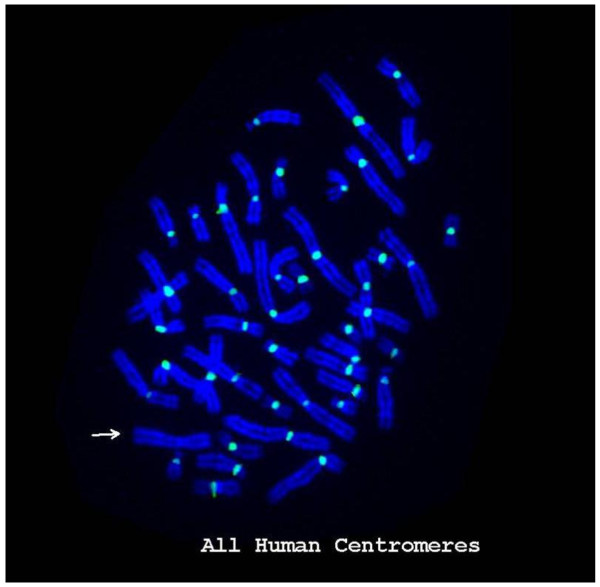
**FISH image of metaphase using a pan-α-satellite probe (green).** Hybridization signals are present on all chromosomes except the abnormal X.

**Figure 4:**
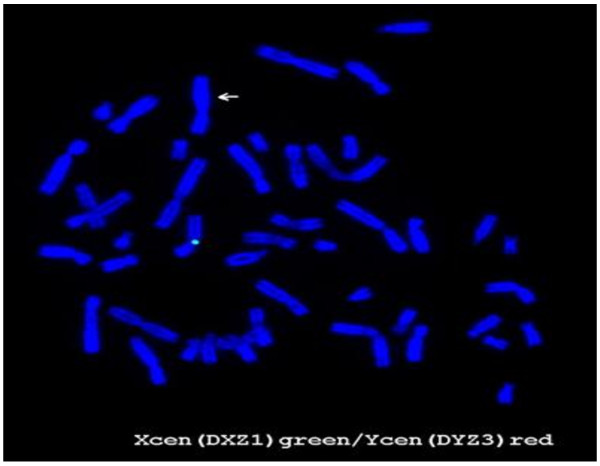
**FISH image of metaphase using X-centromere probe.** The abnormal X chromosome lacks a hybridization signal.

The preliminary FISH analysis revealed lack of hybridization of the abnormal X chromosome with both the X centromere-specific probe and the probe for all human centromeres. This pattern was consistent with absence of an endogenous centromere on the abnormal X chromosome (Figures 3 and 4). This was also confirmed by negative C-banding performed on metaphases with an abnormal X.

**Figure 5:**
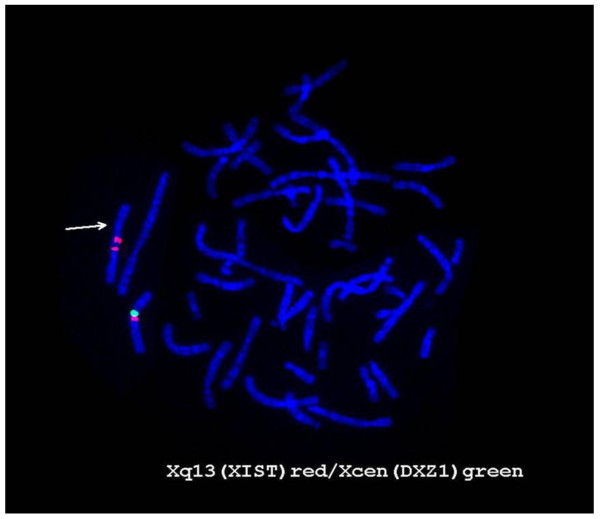
**FISH image of metaphase using XIST gene probe (red).** The abnormal X chromosome has two hybridization signals, which indicates duplication of Xq.

**Figure 6:**
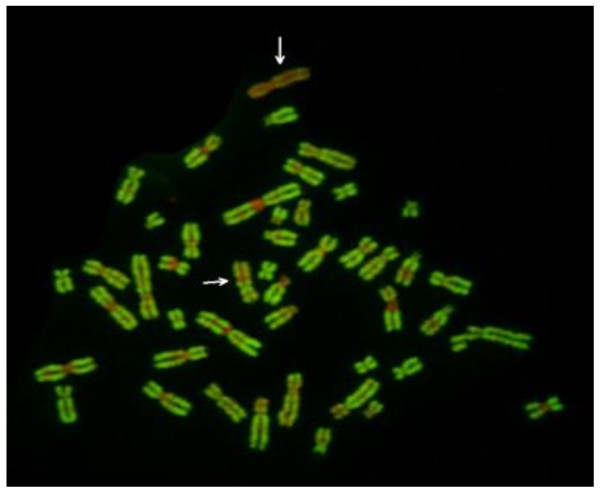
R-banding analysis reveals the abnormal X chromosome to be inactive.

**Figure 7:**
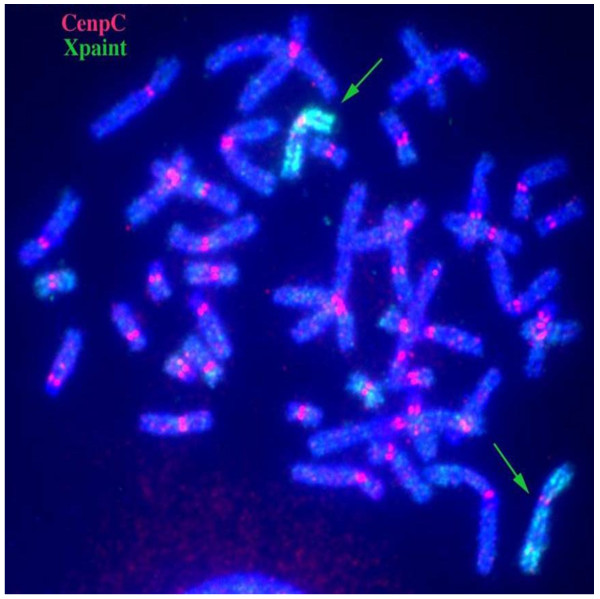
**Centromeric protein C (CENP-C) assay.** CENP-C (red signal) is present on all the chromosomes and defines the presence of a neocentromere in the abnormal X.

Further FISH study for the XIST gene revealed that 2 copies of this gene were present on the abnormal X chromosome (Figure 5). Moreover, the R-banding analysis revealed that the abnormal X chromosome was inactive in all cells (Figure 6). Figure 7 shows the results for combined whole X chromosome painting FISH and simultaneous immunofluorescence staining using anti-CENP-C antibody. The CENP-C assay was positive on both the normal and abnormal X chromosomes, as indicated by the whole X chromosome paint, confirming the presence of a neocentromere in the abnormal X chromosome. The novel position of CENP-C in the abnormal X chromosome defined a neocentromere, which explains its mitotic stability

## Discussion

The incidence of Turner syndrome is approximately 1 in 4000 female live births. Fewer than 5% of Turner syndrome cases are mosaic, with one cell line carrying one normal and one abnormal X chromosome. Our case of mosaic Turner syndrome is the first to be reported with the presence of an isochromosome for Xq, in which a neocentromere is present in lieu of a normal centromere. The karyotype of our patient was thus designated as 46,X,neo(X)(qter- > q12::q12 > q21.2- > neo- > q21.2- > qter)[42]/45,X[8], based on the results obtained from chromosome and FISH analysis.

**Figure 8:**
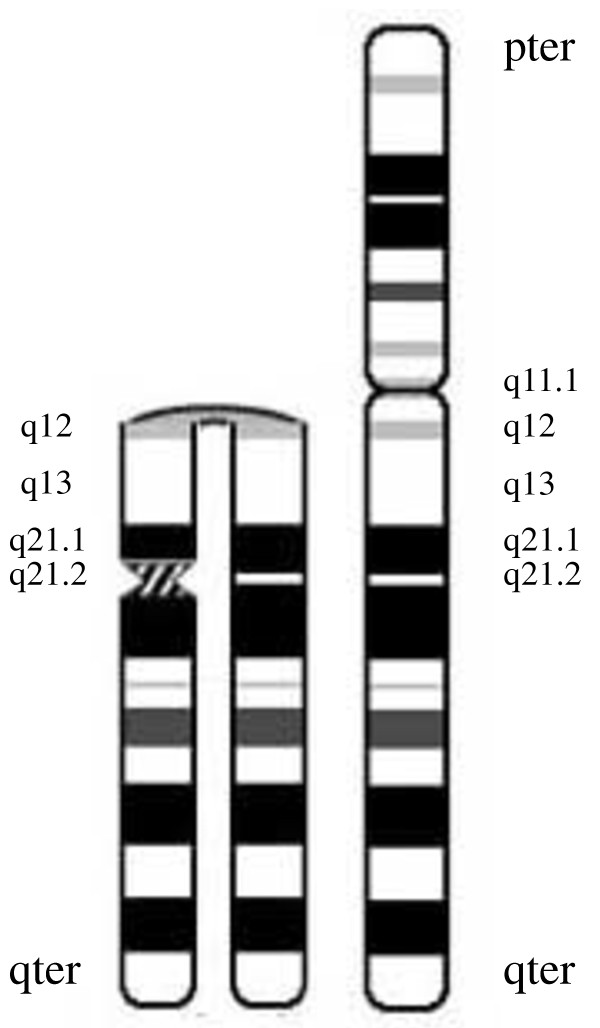
**Ideogram of the neocentric X.** The duplicated copies are mirror images around the breakpoint Xq12. The neocentromeric constriction has occurred within the band Xq21.2 of one of the duplicated regions.

This iso(Xq) essentially represents an inverted duplication of Xq, which possibly occurred at maternal meiosis I; this inversion apparently involves anomalous or U-type crossing over at Xq12 as described by Warburton [4]. The duplicated copies are mirror images around the breakpoint Xq12 (Figure 8). This acentric fragment would usually be lost but has rescued itself by generating a neocentromere, which functions similarly to a normal centromere, at one of the duplicated regions. Lack of a centromere and presence of a neocentromere in the abnormal X chromosome of our patient was confirmed by FISH and CENP-C staining. The novel position of CENP-C and absence of alpha satellite DNA in the abnormal X chromosome defined a neocentromere, which also explains its mitotic stability. The mosaicism observed in the karyotype of our patient is very likely a consequence of gradual formation, stabilization, and functioning of the neocentromere after several post-fertilization cell divisions, during which the iso(Xq) may be lost in a proportion of cells [14,26]. 

X-inactivation is the process by which most genes on one of the two X chromosomes in females are silenced epigenetically. In normal females cells, the choice of which X chromosome (paternal or maternal) is to be inactivated is a random one that is then maintained in each clonal lineage. However, an abnormal X chromosome is always the inactive one if it is structurally unbalanced. This was confirmed in our patient by R-banding, which showed that the abnormal X was inactivated in all metaphases carrying this abnormal chromosome. Presence of an XIST gene, the key master locus for X inactivation, at band Xq13 was also confirmed by FISH: the abnormal X chromosome carried two copies, and the normal X carried one. The XIST gene is normally expressed only from the inactive X and is transcriptionally silent on the active X. The selective inactivation of the abnormal X in our patient likely results in a protective effect from possible abnormalities attributable to it.

## Conclusion

To our knowledge, this is the first case of mosaic Turner syndrome involving an analphoid iso(Xq) chromosome with a proven neocentromere among 90 previously described cases with a proven neocentromere.

## Methods

### Chromosome analysis

A peripheral blood sample from a 15-year-old girl was referred to our laboratory for cytogenetic analysis. Her clinical features were suggestive of Turner syndrome, including short stature and primary amenorrhea. Metaphase chromosome preparations were obtained from the patient and her mother according to standard procedures. Chromosomes were analyzed with G-banding at the resolution level of 550 bands. C-banding was also performed on the patient’s metaphase cells. The father was not available for karyotyping.

### Analysis by Fluorescence in situ hybridization (FISH)

FISH analyses were carried out according to the protocols recommended by the manufacturer of the DNA probes. This study used α-satellite DNA probes for all chromosome centromeres, a mixture of probes for X chromosome centromere (DXZ1) and Y chromosome centromere (DYZ3), a locus specific probe for XIST gene located at Xq13 (spectrum orange)/CEP X centromere (spectrum green) and a probe for whole chromosome painting.

### Analysis by R-Banding

R-banding for evaluation of X-inactivation status was then performed. To obtain RBA (reverse banding by 5-bromodeoxyuridine and acridine orange), the culture techniques were modified by adding 75 μg/mL 5-bromodeoxyuridine 6.5 hours before harvest, and slides were prepared by a modification of the technique of Van Dyke et al. [27].

### Ethical approval and consent

These studies were performed on anonymized samples received in the clinical laboratory and thus were exempted from the requirement for consent by an opinion for the Western Institutional Review Board.

### Analysis by CENP-C staining

To investigate the presence of a neocentric marker chromosome, immunofluorescence analysis with antibody to centromere protein C (CENP-C) was employed on fresh 3:1 methanol:acetic acid fixed chromosomes according to published protocols [18].

## Competing interests

The authors declare that they have no competing interests.

## Authors’ contributions

MH First co-author; drafted and finalized the manuscript. BTW First co-author; initiated the study and finalized the manuscript. PEW conducted the immunofluoresence analysis with antibody to CENP-C. XJY performed R-banding and FISH analyses. FZB made critical comments on the drafted manuscript. MME made comments on the drafted manuscript. AA Corresponding author, final approval of the drafted manuscript. All authors read and approved the final manuscript.
